# Gastrointestinal Involvement in Pediatric Behçet’s Disease: A Focused Analysis from a Comprehensive Systematic Review

**DOI:** 10.3390/diagnostics16193184

**Published:** 2026-09-30

**Authors:** Dimitri Poddighe, Donato Rigante, Micol Romano, Zaure Mukusheva, Nicola Ughi, Erdal Sag, Selcan Demir, Natalie Zitoun, David Piskin, Erkan Demirkaya

**Affiliations:** 1 College of Health Sciences, VinUniversity, Hanoi 100000, Vietnam; 2 Vinmec International Hospital, Hanoi 100000, Vietnam; 3 Innovation in Health Sciences (IHS), VinUniversity, Hanoi 100000, Vietnam; 4 Department of Life Sciences and Public Health, Fondazione Policlinico Universitario A. Gemelli IRCCS, 00168 Rome, Italy; donato.rigante@unicatt.it; 5 Università Cattolica del Sacro Cuore, 00168 Rome, Italy; 6 Division of Pediatric Rheumatology, Department of Pediatrics, Schulich School of Medicine & Dentistry, University of Western Ontario, London, ON N6A 5W9, Canadaerkan.demirkaya@lhsc.on.ca (E.D.); 7 Canadian Behcet’s and Autoinflammatory Diseases Center, University of Western Ontario, London, ON N6A 5W9, Canada; 8 Pediatric CAD, Mother and Child Center, “University Medical Center” Corporate Fund, 010000 Astana, Kazakhstan; 9 Division of Rheumatology, Multispecialist Medical Department, ASST Grande Ospedale Metropolitano Niguarda, 20162 Milan, Italy; 10 Department of Pediatric Rheumatology, Faculty of Medicine, Hacettepe University, 06100 Ankara, Turkey; 11 Department of Pediatric Rheumatology, Faculty of Medicine, Eskisehir Osmangazi University, 26480 Eskisehir, Turkey; selcandemir@yahoo.com; 12 London Health Sciences Centre Research Institute, London, ON N6A 5W9, Canada; 13 Department of Epidemiology and Biostatistics, Schulich School of Medicine & Dentistry, University of Western Ontario, London, ON N6G 2M1, Canada

**Keywords:** Behçet’s disease, pediatric, children, gastrointestinal involvement, systematic review, inflammatory bowel disease, endoscopy, anti-TNF, adalimumab

## Abstract

**Background**: Gastrointestinal (GI) involvement is a clinically relevant but still incompletely characterized manifestation of pediatric Behcet’s disease (PedBD). This systematic review provides a focused analysis of GI involvement in PedBD (addressing its frequency, clinical symptomatology, anatomical/endoscopic features, and therapeutic management) as a dedicated sub-analysis of a broader systematic review. **Methods**: A systematic search of MEDLINE, EMBASE, CINAHL, CENTRAL, and ClinicalTrials.gov was conducted, following PRISMA 2020 guidelines. Observational studies reporting GI involvement in patients with PedBD (disease onset before 16 years of age) were included, and data on frequency, symptoms, anatomical/endoscopic findings, and treatment of GI-PedBD were extracted and synthesized. **Results**: Thirty studies were included. Most studies were not focused on GI manifestations in PedBD as a (primary) study aim. The frequency of GI involvement ranged widely across the selected articles with a median of approximately 15% among studies with ≥50 pediatric BD patients. Abdominal pain and diarrhea were the most consistently reported symptoms. Endoscopic data indicated frequent involvement of the ileocecal region and terminal ileum, with mucosal ulcers and erosions as the predominant findings. Therapeutic data specific to GI-PedBD were limited to two studies only. As an appendix to the systematic review in the discussion, relevant case reports were described: anti-TNF agents, particularly adalimumab, showed favorable outcomes in refractory cases. **Conclusions**: GI involvement in PedBD is not negligible and can be severe. Its clinical and endoscopic characterization remains incomplete due to substantial heterogeneity in reporting practices across studies. Anti-TNF agents may represent an effective therapeutic option, especially for refractory GI-PedBD. Prospective and multicenter studies with standardized GI assessment protocols are needed to better characterize this clinically relevant subgroup of PedBD patients.

## 1. Introduction

Behçet’s disease (BD) is a systemic vasculitis originally described by the Turkish dermatologist Hulusi Behçet in 1937; it is mostly characterized by the occurrence of recurrent oral aphthous ulcers, genital ulcers, and uveitis, although virtually any organ system may be affected, including the skin, joints, central nervous system, and gastrointestinal tract [1,2]. Although BD is characterized by a peak onset in the third and fourth decades of life, and its prevalence varies according to genetic and geographical factors, the disease has been increasingly recognized in the pediatric population. Pediatric BD (PedBD) is generally defined by clinical onset before the age of 16 years and represents approximately 4–26% of all BD cases across different studies [3,4,5,6].

Gastro-intestinal (GI) involvement in BD encompasses a broad spectrum of manifestations, ranging from non-specific symptoms such as abdominal pain and diarrhea to severe complications including intestinal perforation, hemorrhage, and fistula formation [7,8]. The pathological mark of GI-BD is represented by deep, punched-out ulcerations, most frequently localized in the ileocecal region in adult series, although any segment of the GI tract may be involved [9]. The differential diagnosis with inflammatory bowel diseases (IBDs), particularly Crohn’s disease, remains a significant clinical challenge [10]. In the pediatric setting, GI-BD has been reported variably across different cohorts, with frequencies ranging widely depending on the study population, research objectives, and method of GI assessment. Moreover, several classification criteria for BD have been developed and applied across available studies, including the International Study Group criteria (ISG), the International Criteria for Behçet’s Disease (ICBD), and the Pediatric Behçet’s Disease classification criteria (PEDBD criteria) [11].

Despite being a potentially severe manifestation that may complicate the clinical course and worsen the prognosis of PedBD, a comprehensive and systematic characterization of GI involvement in this age group is still lacking. This systematic review aimed to provide the first comprehensive synthesis of available evidence on GI involvement in PedBD, with a focus on: (i) the frequency of GI manifestations across published cohorts; (ii) the clinical symptomatology; (iii) the anatomical distribution and endoscopic/pathological features of GI lesions; and (iv) the therapeutic approaches specifically adopted for GI-PedBD.

## 2. Methods

### 2.1. Study Design

This systematic review was conducted and reported in accordance with the Preferred Reporting Items for Systematic Reviews and Meta-Analyses (PRISMA 2020) guidelines [12].

### 2.2. Eligibility Criteria

Studies were eligible for inclusion if they: (i) reported original data on PedBD (defined as disease onset before the age of 16 years, or explicitly labeled as juvenile/pediatric BD); (ii) reported data on GI involvement as a distinct clinical manifestation; (iii) were published as full-text articles in English; and (iv) were original research studies (cohort, cross-sectional, registry-based, or case-series studies with ≥5 patients).

Exclusion criteria were: (i) adult-only populations without separate reporting of pediatric data; (ii) case reports; (iii) conference abstracts, letters, and editorials; (iv) review articles; and (v) studies with aggregated adult/pediatric data that could not be disaggregated.

### 2.3. Search Strategy

A comprehensive literature search was conducted up to 17 December 2024, across the following databases: MEDLINE (via Ovid, 1946–present), EMBASE (via Ovid, 1947–present), CINAHL Complete (via EBSCOhost), Cochrane Central Register of Controlled Trials (CENTRAL), and ClinicalTrials.gov. The complete search strategies for each database are provided in Appendix A (Table A1), which are the same as those used in our main and general systematic review on the diagnosis, treatment and monitoring of PedBD, [13] of which this analysis represents a subtask. Search terms combined controlled vocabulary (MeSH/Emtree headings) and free-text keywords for Behçet’s disease and pediatric/child populations. No date restrictions were applied.

### 2.4. Study Selection

Each retrieved article from the aforementioned general and systematic literature search was re-screened according to title and abstract by two independent reviewers for eligibility. Then, eligible articles were evaluated from full texts by two reviewers (DP and DR), and any discrepancies were resolved by a third reviewer (MR).

Specifically, the full-text articles were selected if they could provide data about one or more of the following research questions: (i) the frequency of GI involvement in PedBD; (ii) the clinical manifestations of GI-PedBD; (iii) the anatomical distribution and/or endoscopic/pathological features of lesions in GI-PedBD; and (iv) therapeutic approaches specifically adopted for GI-PedBD patients.

### 2.5. Data Extraction

For each research question, specific data extraction forms were developed. Overall, the following variables were collected: first author, publication year, country, study aim, study design, study period, specific study population (e.g., all PedBD patients or specific subgroups of PedBD), size of study population, sex distribution, age at PedBD onset, diagnostic criteria used, number of patients with GI involvement, GI involvement frequency, GI symptoms reported, anatomical location of GI lesions, endoscopic/pathological findings of GI lesions, and specific therapeutic interventions for GI-PedBD.

### 2.6. Quality Assessment

The Joanna Briggs Institute (JBI) critical appraisal tools were used to assess the methodological quality of the included studies according to study design (https://jbi.global/critical-appraisal-tools, accessed on 10 September 2024). Two independent reviewers (NU and DPi) performed the assessments, with disagreements resolved through discussion. Each JBI item was rated as low concern, some concerns, or high concern. Based on the overall appraisal, studies with up to three items rated as some concerns or high concern were classified as having a low risk of bias, four as having a moderate risk of bias, and more than four as having a high risk of bias (Appendix B).

### 2.7. Data Synthesis

Due to the substantial heterogeneity in study design, patient populations, diagnostic criteria, and GI assessment methods, a formal meta-analysis was not performed. Instead, data were synthesized narratively, organized according to the aforementioned research questions.

## 3. Results

**Figure 1:**
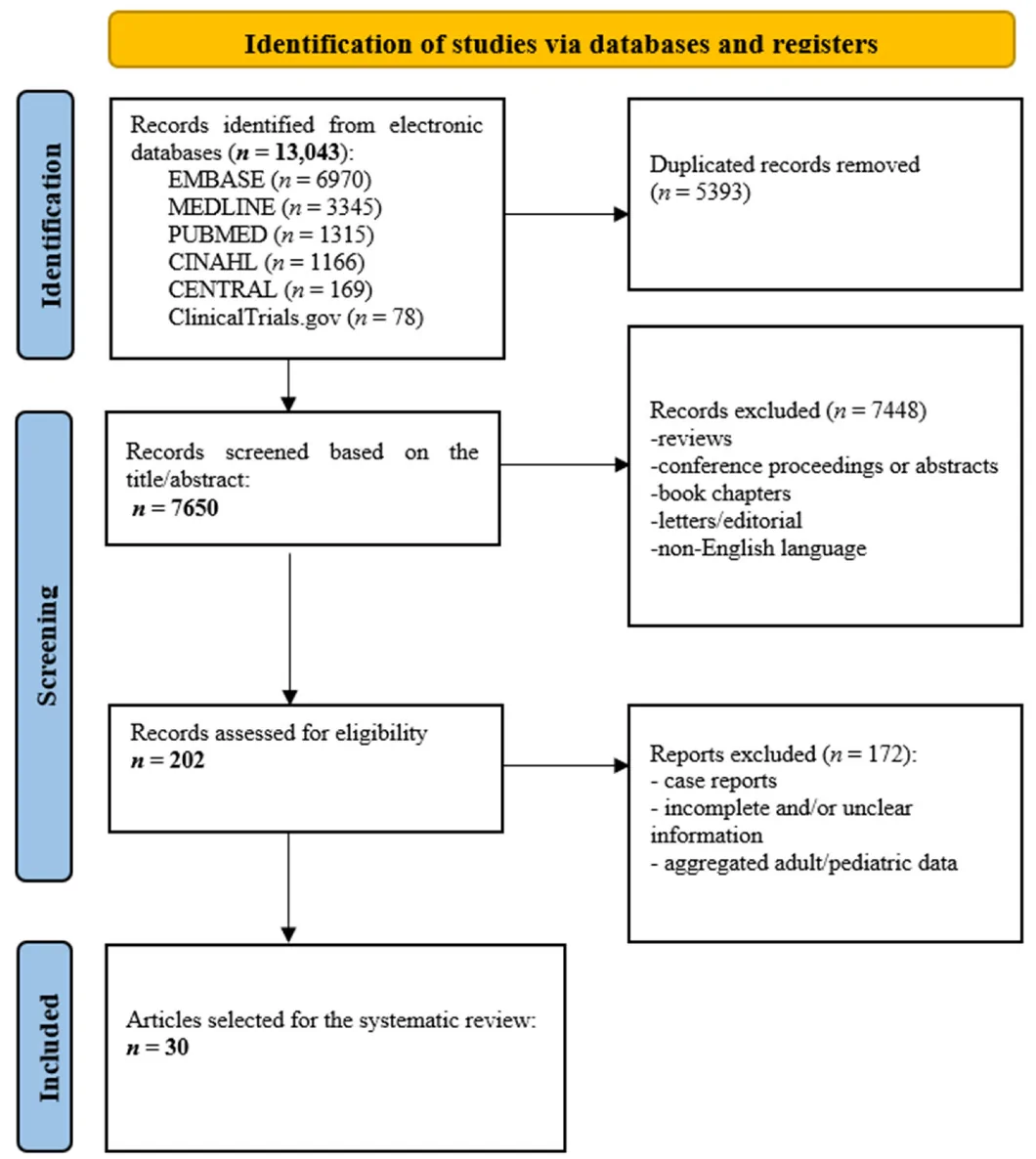
PRISMA 2020 flowchart.

The search yielded a total of 13,043 records (EMBASE: *n* = 6970; MEDLINE: *n* = 3345; PubMed: *n* = 1315; CINAHL: *n* = 1166; CENTRAL: *n* = 169; ClinicalTrials.gov: *n* = 78). After the removal of 5393 duplicates, 7650 records were screened based on title and abstract. Records not meeting the eligibility criteria (reviews, conference proceedings, book chapters, letters/editorials, non-English language publications) were excluded (*n* = 7448), leaving 202 records for full-text assessment. Of these, 172 reports were excluded (case reports; incomplete and/or unclear information; aggregated adult/pediatric data). A total of 30 articles were ultimately selected for the systematic review. The complete selection process is illustrated in Figure 1 (PRISMA 2020 flow diagram).

### 3.1. Study Characteristics and Included Studies

A total of 30 studies met the inclusion criteria and were included in the systematic review [14,15,16,17,18,19,20,21,22,23,24,25,26,27,28,29,30,31,32,33,34,35,36,37,38,39,40,41,42,43]. The included studies were published between 1998 and 2024 (spanning a 26-year period) and originated from a wide range of geographic settings: mainly, European and Asian countries. The vast majority of studies were retrospective in design. The diagnostic criteria applied were heterogeneous and included the ISG criteria, the ICBD criteria, the PedBD classification criteria, the Japanese BD Research committee, and various combinations thereof.

### 3.2. Frequency of GI Involvement in Pediatric BD

As summarized in Table 1 [14,15,16,17,18,19,20,21,22,23,24,25,26,27,28,29,30,31,32,33,34,35,36,37,38,39,40,41], the frequency of GI involvement ranged widely, from 0.6% (in a Turkish cohort of 159 juvenile BD patients compared to adult-onset forms) [36] to 85.7% (in a small Italian cohort of seven children with neuro-BD, where GI data were incidental to the primary study aim) [17]. Indeed, one study (by Ostrovsky et al.) explicitly reported no children with GI involvement in their cohort of 25 PedBD patients; notably, this study aimed to investigate ocular BD according to age at disease onset [35]. Excluding this last study and those studies that focused on specific subgroups of PedBD patients (e.g., PedBD with neurological complications, PedBD with musculoskeletal complaints, PedBD with uveitis, PedBD with renal hypertension) [17,32,35,39,41], the frequency ranged from 0.6% to 56.5%.

The highest GI frequencies were reported in the aforementioned UK cohort studied by Nathanpisal et al. (56.5%; *n* = 26/46) [22], and in a multicenter Italian cohort (42.7%; *n* = 47/110) reported by Gallizzi et al. [23]. Intermediate frequencies were observed in several other cohorts, including a French international cohort (29.5%; *n* = 46/156) [21], a Taiwanese cohort (29.1%; *n* = 16/55) [25], and two Chinese cohorts (33.1%, *n* = 82/248; 29.0%; *n* = 20/69) [29,38]. Conversely, lower frequencies were found in large Turkish cohorts (3.6%; *n* = 8/225) 4.8%; *n* = 4/83) [19,31].

When considering only studies with ≥50 pediatric BD patients and a general (not organ-specific) study aim (*n* = 16 studies) [14,19,20,21,23,24,25,26,27,28,29,30,31,36,37,38], the median GI involvement frequency was approximately 15%, with an interquartile range of approximately 6–29%.

### 3.3. Gastro-Intestinal Symptoms in PedBD

Seven studies provided detailed reporting of GI symptoms in PedBD (Table 2) [14,18,22,30,32,42,43]. Abdominal pain was the most consistently reported symptom, documented in all seven studies. Diarrhea was the second most frequent symptom, reported in all seven studies. Gastric upset (with dyspepsia-like symptoms) was reported in three patients by Koné-Paut et al. [14]; nausea, reported as part of an aggregated symptom category (abdominal pain/nausea/diarrhea), was reported by Gaggiano et al. [32]. More severe GI manifestations were documented in the UK cohort by Nanthapisal et al. [22], including melena, hematemesis, and anal/perianal ulcers. Vomiting was also reported in one study [43].

**Table 1:** Characteristics of included studies and frequency of gastrointestinal involvement in pediatric Behçet’s disease.

Ref.	First Author	Year	Country	Study Aim	Study Design	Study Period	BD Study Population	N M:F	Age at BD Onset	BD Diagnostic Reference	GI PedBD Patients (M:F)	GI Involvement (Definition)	Notes
[14]	Koné-Paut	1998	France	To characterize the clinical picture of BD in children	Retrospective	n/a	PedBD (*n* = 65)	33:32	9.6 y (me)	ISG	9 (5:4)	13.8% (C:9) (E:4)	Questionnaire completed by five international BD specialists
[15]	Krause	1999	Israel	To compare the clinical spectrum of childhood-onset BD with adult-onset disease	Retrospective	n/a	PedBD (*n* = 19)	11:8	6.9 y (m)	ISG	7 (n/a)	36.8% (C)	
[16]	Vaiopoulos	1999	Greece	To describe the clinical features of juvenile BD	Retrospective	n/a	PedBD (*n* = 18)	11:7	10.3 y (m)	ISG	2 (n/a)	11.1% (n/a)	
[17]	Vignola	2001	Italy	To study brain perfusion SPECT in juvenile neuro-BD patients *	Retrospective	n/a	NeuroBD (*n* = 7)	7:0	9.1 y (m)	ISG	6:0	85.7% (C)	* study population restricted to neuro-BD
[18]	Borlu	2006	Turkey	To determine clinical features and outcome of juvenile BD	Retrospective	1997–?	PedBD (*n* = 17)	12:5	7 y (m)	ISG	2 (n/a)	11.8% (C)	
[19]	Karıncaoğlu	2008	Turkey	To describe demographic and clinical features of juvenile BD vs. adults	Retrospective	n/a	PedBD (*n* = 83)	38:45	12.3 y (m)	NS	4 (n/a)	4.8% (E)	
[20]	Koné-Paut	2011	France	Expert committee classification of PedBD	Retrospective & Prospective Registry	?–2010	PedBD (*n* = 110)	56:54	8.2 y (me) 8.1 y (m)	PEDBD	28 (n/a)	25.5% (C)	Based on data from 11 countries in Europe, North Africa and the Middle East
[21]	Koné-Paut	2016	France	To establish a PedBD prospective cohort to set classification criteria	Prospective	n/a	PedBD (*n* = 156)	M:F ≃1	8.9 y (m)	PEDBD	46 (n/a)	29.5% (C)	Based on an international expert consensus group from 12 countries
[22]	Nanthapisal	2016	UK	To describe clinical spectrum, treatment and outcome of juvenile BD	Retrospective	1987–2012	PedBD (*n* = 46)	22:24	4.9 y (me)	ISG, ICBD	26 (n/a)	56.5% (C:26) (E:8)	
[23]	Gallizzi	2017	Italy	To collect demographic, clinical and therapeutic data from pediatric patients	Retrospective	n/a	PedBD (*n* = 110)	62:48	8.3 y (m)	ISG, ICBD, PEDBD	47 (n/a)	42.7% (C)	PedBD patients enrolled in the Eurofever registry by the Italian Pediatric Rheumatology Centers
[24]	Shahram	2018	Iran	To report characteristics of juvenile BD	Retrospective	1975–2016	PedBD (*n* = 204)	103:101	10.5 y (m)	ICBD	12 (5:7)	5.9% (C)	
[25]	Hu	2019	Taiwan	To evaluate clinical characteristics and anti-TNF therapy in PedBD	Retrospective	2008–2017	PedBD (*n* = 55)	22:33	11 y (me)	ICBD, PEDBD	16 (n/a)	29.1% (C)	
[26]	Butbul-Aviel	2020	Israel	To analyze the main characteristics of PedBD patients	Retrospective	2014–2019	PedBD (*n* = 205)	107:98	11.1 y (me)	ISG, ICBD, PEDBD	27 (n/a)	13.2% (C)	Patients from Turkey and Israel
[27]	Gezgin Yıldırım	2020	Turkey	To determine outcome of juvenile BD	Retrospective	2008–2018	PedBD (*n* = 57)	33/24	10 y (me)	ISG, ICBD, PEDBD	5 (n/a)	8.8% (C)	
[28]	Pain	2021	UK	To define incidence and prevalence of BD in children	Prospective	2015–2017 *	PedBD (*n* = 56)	24:32	6.3 y (me)	ICBD	9 (7:2)	16.7% (C)	* data analysis period: Surveillance study supported by the British Paediatric Surveillance Unit for the study of rare conditions, established since 1986.
[29]	Zou	2021	China	To determine the clinical phenotype of juvenile BD	Retrospective	2012–2020	PedBD (*n* = 69)	32:37	12 y (me)	ISG	20 (10:10)	29.0% (n/a)	
[30]	Ekici-Tekin	2022	Turkey	To determine clinical/therapeutic aspects of PedBD	Retrospective	2007–2020	PedBD (*n* = 72)	32:40	11 y (me)	ISG, ICBD, PEDBD	15 (n/a)	20.8% (C:15) (E:2)	
[31]	Demir	2023	Turkey	To determine the main characteristics of PedBD	Retrospective	2015–2020	PedBD (*n* = 225)	109:116	10.9 y (me)	ISG, Japanese, PEDBD	8 (n/a)	3.6% (n/a)	
[32]	Gaggiano	2023	Italy	To focus on musculoskeletal manifestations in children with BD	Retrospective	n/a	PedBD with MSM (*n* = 141) *	n/a	10 y (me)	ISG, ICBD, PEDBD	13 (n/a)	9.2% (C:13) (E:6)	AIDA Network BD Registry by 13 international centers. * study population restricted to PedBD with MSM
[33]	Güngörer	2023	Turkey	To compare PedBD patients with/without thrombosis	Retrospective	2010–2022	PedBD (*n* = 85)	39:46	10.1 y (m)	ISG, ICBD, PEDBD	19 (n/a)	22.4% (C)	
[34]	Mastrolia	2023	Italy	To match clinical and treatment features of children *versus* adults with BD	Retrospective	?–2021	PedBD (*n* = 33)	19:14	12 y (me)	ISG, ICBD, PEDBD	8 (n/a)	24.2% (C)	
[35]	Ostrovsky	2024	Israel	To investigate ocular BD according to age at disease onset	Retrospective	2000–2021	PedBD (*n* = 25)	20:5	12.7 y (m)	NS	0	0% (E)	
[36]	Boluk	2024	Turkey	To determine characteristics and systemic involvement of late-onset BD	Retrospective	n/a	PedBD (*n* = 159)	61:98	13 y (me)	ISG, ICBD	1 (n/a)	0.6% (E)	
[37]	Bozkurt	2024	Turkey	To determine clinical course of pediatric-onset BD into adulthood	Retrospective	n/a	PedBD (*n* = 93)	60:33	n/a	ISG	6 (4:2)	6.5% (n/a)	
[38]	Hu	2024	China	To define the main characteristics of pediatric patients with BD	Retrospective	2012–2023	PedBD (*n* = 248)	n/a	12 y (me)	ISG, ICBD, PEDBD	82 (n/a)	33.1% (E)	
[39]	Maccora	2024	Italy	To describe ocular outcomes/treatment response of PedBD with uveitis	Retrospective	?–2023	PedBD uveitis (*n* = 97) *	n/a	11.5 y (me)	ICBD, ISG, PEDBD	4 (n/a)	9.1% (C)	* study population restricted to the specific PedBD with uveitis
[40]	Mastrolia	2024	Italy	To report features/outcomes of PedBD with thrombosis	Retrospective	n/a	PedBD (*n* = 111)	37:84	12.8 y (me)	ISG, ICBD, PEDBD	34 (n/a)	30.6% (C)	Eurofever registry (patients form Italy, Greece, Austria, Spain and Turkey)
[41]	Wang	2024	China	To investigate hypertension secondary to renal artery lesions in PedBD	Retrospective	2009–2022	PedBD with renal hypertension (*n* = 65) *	n/a	9 y (me)	PEDBD	3 (n/a)	50% (E)	* study population restricted to the specific PedBD subgroup developing renal hypertension

Abbreviations: m: mean; me: median; PedBD: pediatric Behçet; n/a: information not available; N: number; M: male; F: female; y: years; C: clinical definition of PedBD; E: endoscopic definition of PedBD; *: specification related to the information reported in the same article/row.

**Table 2:** Gastrointestinal symptoms in pediatric Behçet’s disease: summary of included studies.

First Author [Ref]	Year, Country	GI-PedBD Patients (N)	Abdominal Pain (N)	Diarrhea (N)	Melena/ Hematochezia (N)	Vomiting (N)	Dyspepsia (N)	Other GI Complaints (N)	Notes
Koné-Paut [14]	1998 France	9	6	3	-	-	3 *	Hepatomegaly: 1 Splenomegaly: 1	* “gastric upset”
Borlu [18]	2006 Turkey	2	2	2	-	-	-	-	.
Metreau-Vastel [42]	2010 France	6	6	6	-	-	-	-	Study focused on neuro-BD; GI data incidental. 1 patient had severe ulcerative-colitis-like features.
Nanthapisal [22]	2016 UK	26	12	3	2	-	-	Recurrent anal/perianal ulcers: 5	
Ekici-Tekin [30]	2022 Turkey	15	15	2 *	-	-	-	-	* These 2 patients had bloody diarrhea
Gaggiano [32]	2023 Italy	13	12 *		-	-	*	-	* Data reported aggregated as ‘abdominal pain/nausea/diarrhea’ in the source
Lv ^†^ [43]	2024 China	24	17	2	4 *	4	-	-	* All 4 patients had hematochezia. ^†^ This study enrolled exclusively GI-PedBD patients with early-onset and atypical presentations.

Abbreviations: PedBD: pediatric Behçet; GI: gastrointestinal: n/a: information not available; N: number; Ref: reference number; *^,†^: specification related to the information reported in the same article/row.

### 3.4. Anatomical Location and Endoscopic Features of Gastrointestinal Lesions in PedBD

Detailed information on the anatomical location and endoscopic/histopathological features of GI lesions was available in only four studies, reflecting the limited use of systematic endoscopic evaluation in PedBD cohorts (Table 3) [22,32,38,43].

Among the limited pediatric cohorts in which endoscopic assessment was available, ileocecal and terminal ileal involvement were the most frequently reported locations, documented in the two largest endoscopic series (Hu et al.: ileocecal *n* = 49/82, 59.8%; Lv et al.: ileum/ileocecal *n* = 14/24, 58.3%) [38,43]. The esophagus and stomach were the next most commonly involved sites in the study by Nanthapisal et al. [22], but the number of patients undergoing endoscopy was definitely lower than in the previous studies mentioned above. Ulcers and erosions were the predominant endoscopic finding, along with general inflammatory changes of the intestinal mucosa. In the study by Lv et al. [43], although all 24 patients satisfied established BD classification criteria (ICBD and/or PEDBD criteria) at the time of diagnosis, the research was specifically designed to characterize a selected subset of GI-PedBD patients with earlier disease onset (median age 4.0 years) and/or atypical clinical features. Indeed, this study population included children eventually diagnosed with monogenic autoinflammatory syndromes mimicking BD (identified in 7 of 18 genetically tested patients). However, this study provides interesting insights in terms of the disease location in PedBD patients, since most patients were assessed via GI endoscopy.

### 3.5. Therapeutic Management of GI-PedBD

Specific therapeutic data for GI manifestations in PedBD were reported in only two studies included in the main analysis [25,37]. In a Taiwanese retrospective cohort of 55 PedBD patients (16 with GI involvement, 29.1%) studied by Hu et al. [25], treatment effectiveness was examined for corticosteroids, colchicine, and anti-TNF agents. The study demonstrated that patients with GI symptoms, younger age at disease onset, and higher serum C-reactive protein levels at baseline tended to have a poorer response to conventional treatment strategies (including corticosteroids and colchicine), supporting the use of anti-TNF agents (particularly infliximab and adalimumab) as an effective second-line option in refractory GI-PedBD. In a Turkish retrospective cohort of 93 pediatric-onset BD patients followed into young adulthood by Bozkurt et al. [37], GI involvement was reported in 6 patients; treatment included corticosteroids and immunosuppressants.

**Table 3:** Gastrointestinal pathological involvement in pediatric Behçet’s disease: summary of included studies.

First Author	Year, Country	GI-PedBD Patients (N)	Endoscopy (N)	Anatomical Location (N)							Type of Lesions (N)		Notes
				*Esophagus*	*Stomach*	*Duodenum/* *Small Bowel*	*Ileum*	*Ileo-Cecal*	*Colon*	*Rectum*	*Ulcers and* *Erosions*	*Inflammation (Non-Ulcerative)*	
Nanthapisal [22]	2016 UK	26	10	2	3	-	-	-	2	-	2 (colon) 1 (esophagus)	3 (gastritis); 1 (esophagitis)	
Gaggiano [32]	2023 Italy (international)	13	8	n/a							2	6	- Anatomical location not specified. - endoscopy not conducted: 5; negative: 2
Hu [38]	2024 China	82	82	20		9	-	49	21	-	82	Not specified	
Lv [43]	2024 China	24	24	3	4	4	14	14	5	2	20	6	- Pan-enteric involvement noted. - Pseudo-polyps around ileocecal ulcers in 4 patients

Abbreviations: PedBD: pediatric Behçet; GI: gastrointestinal: n/a: information not available; N: number.

## 4. Discussion

This systematic review provides the first comprehensive synthesis of available evidence on GI involvement in PedBD, analyzing data from 30 studies published over a 26-year period (1998–2024) and encompassing around 3000 PedBD patients from diverse geographic settings. Our findings confirm that GI involvement is a clinically relevant and not uncommon manifestation of BD in children and adolescents, although its reported frequency varies substantially across published cohorts. These findings may have relevant implications for the overall clinical management and damage accrual in children with BD [44,45].

The wide variability in GI frequency (0% to 85.7%) observed across studies reflects the profound heterogeneity of the available literature, which can be explained by several factors.

First, the use of different diagnostic criteria for BD introduces systematic differences in patient selection and disease classification.

Second, the variability in the definition and assessment of GI involvement is critical: while some studies applied systematic endoscopic evaluation, many relied on clinical symptom reporting alone. In this regard, the included studies did not adopt a consistent operational definition of “GI involvement”, ranging from the mere report of GI symptoms without objective confirmation, to suspected involvement based on clinical and/or laboratory findings, to objectively confirmed GI disease based on endoscopy, imaging, or histology. Although an absolute and uniform operational definition could not be retrospectively applied across the heterogeneous set of included studies, we believe this systematic review can help raise this important point for future studies (where an explicit and/or graded definition of GI involvement, distinguishing symptomatic, suspected, and objectively confirmed disease, should be adopted). Unfortunately, consolidated consensus criteria for diagnosing gastrointestinal BD, especially in the pediatric population, do not currently exist, although some groups (e.g., the Korean IBD Study Group) have proposed a set of diagnostic criteria for intestinal BD in adults [46,47].

Third, geographic and ethnic differences in BD phenotype, which are described in adult patients [44,48,49], likely contribute to the variation also observed in pediatric series. When restricting the analysis to studies with a general study aim and a sufficiently large sample size (≥50 patients), a more consistent GI frequency emerges, with a median of approximately 15% and most estimates falling between 6% and 29%. This range is broadly consistent with published adult BD data, where GI involvement is reported in approximately 10–50% of patients depending on the geographic context and ascertainment method [50,51,52]. Notably, the UK cohort reported by Nanthapisal et al. [22] reported one of the highest frequencies (56.5%), which may reflect a referral bias (tertiary center study) as well as the more systematic use of endoscopic investigation in that cohort.

The clinical symptomatology of GI-PedBD, as synthesized from seven studies providing detailed symptom data [14,18,22,30,32,42,43], is dominated by abdominal pain and diarrhea, and thus by symptoms that are non-specific and shared with a broad differential diagnosis including IBDs, particularly Crohn’s disease. This diagnostic overlap represents a major clinical challenge, especially in the pediatric setting, where BD may not be immediately suspected in a child presenting with intestinal inflammation [53]. The reported occurrence of more severe manifestations, including melena, hematemesis, hematochezia, and anal/perianal ulcers, underscores that GI-PedBD may follow a severe and potentially life-threatening course, warranting early recognition and prompt intervention. Even though a comparison between studies of pediatric and adult patients was beyond the scope of this pediatric-focused systematic review, abdominal pain and diarrhea are also the most commonly reported symptoms in adult series [9,50,51,54]. It should also be emphasized that the existing literature on GI-PedBD may overemphasize symptomatic presentations: in adult BD, gastrointestinal ulcers (particularly in the ileocecal region) could frequently be asymptomatic prior to definitive diagnosis, with disease sometimes recognized only after an acute abdominal event such as an appendicitis-like presentation, perforation, or hemorrhage. Indeed, in a large cross-sectional study of 1232 adult BD patients undergoing endoscopy, 22.1% had GI ulcers, of whom 61.5% were asymptomatic at presentation [55]. Although comparable data are lacking in the pediatric population, this observation suggests that a purely symptom-based approach may underestimate the true burden of GI involvement in PedBD as well.

Based on the limited endoscopic data available, the anatomical distribution of GI lesions in PedBD shows involvement of the ileocecal region and terminal ileum as the predominant sites, followed by the colon, esophagus, and stomach as the most common sites, which is consistent with the pattern described in adult intestinal BD [54,56]. However, the current analysis underscores the pan-enteric potential of GI-PedBD, including gastric and esophageal involvement.

As previously noted, the differential diagnosis between IBDs (especially Crohn’s Disease) and PedBD with predominant GI involvement can be challenging. The limited number of pediatric studies on this topic does not allow us to make any clear comparison between these diseases in terms of clinical characteristics, disease location and features of intestinal lesions, as has been achieved in some studies with adult patients, which reported some differential characteristics. For instance, in a large series of 250 patients, Lee et al. suggested that round-shaped ulcers with focal distribution favored GI-BD diagnosis, whereas segmental or diffuse lesions were more characteristic of Crohn’s disease [57]. Some differences in endoscopic parameters such as ulcer number, size, shape and anorectal involvement could contribute to distinguishing the two conditions inside an integrated model, according to a study by Zhang et al. [58] However, these endoscopic distinguishing features have been derived predominantly from adult cohorts and cannot automatically be extrapolated to pediatric disease, for which dedicated comparative studies are still lacking. Beyond endoscopic appearance, BD and Crohn’s disease also differ substantially in their overall clinical presentation and diagnostic framework. BD is a multisystem, variable-vessel vasculitis in which recurrent oral ulcers, genital ulcers, and skin lesions represent the core clinical features, while GI involvement is one of several possible visceral manifestations; the diagnosis remains primarily clinical, with endoscopy serving mainly to confirm the GI phenotype [1]. Crohn’s disease, in contrast, is a primary GI disorder in which intestinal symptoms are almost universally present at diagnosis, whereas mucocutaneous manifestations are uncommon; genital ulcers, in particular, are absent [59], in contrast with BD, in which nearly all patients have recurrent oral ulcers and approximately 70% have genital ulcers [60]. Crohn’s disease also shows distinct endoscopic morphology, distribution patterns, and complication profiles compared with intestinal BD [61]. Distinguishing GI-PedBD from inflammatory bowel diseases, particularly Crohn’s disease, remains difficult in pediatric practice.

Therapeutic data specific to GI-PedBD are strikingly limited, with only two studies from the main analysis providing meaningful information on treatment outcomes [25,37].

Therefore, as a supplementary narrative case-based review (separate from the systematic review synthesis), we further retrieved and examined individual GI-PedBD patients described in case reports and small case series, in order to mitigate the shortage of studies providing precise information on the therapy of PedBD and to gain further insight into the therapeutic management of these patients.

All of these patients are summarized in Supplementary Table S1 [62,63,64,65,66,67,68,69,70,71,72,73,74,75,76,77]. In total, 21 individual GI-PedBD patients were described in case reports that we could retrieve up to the end date of this systematic review. These cases span a broad age range (from one month of life until sixteen years of age, in terms of onset of GI disease) and include patients from several countries (Canada, the United Kingdom, the United States, Japan, Italy, Taiwan, Turkey, and China).

From a therapeutic standpoint, this case-based narrative review collectively illustrates the progressive evolution of treatment strategies for GI-PedBD over three-four decades. Earlier cases (before 2000) were predominantly managed with corticosteroids, sulfasalazine, and, when available, azathioprine, with surgical resection required in several refractory cases [62,63,64,65,66]. Across this four-decade span, surgery was employed both as an emergency intervention for acute complications (e.g., intestinal perforation, hemorrhage, or obstruction) and as an elective procedure for disease that remained medically refractory despite conventional treatment; notably, the majority of these surgical cases predate the availability of biologic therapies, and the need for surgery appears to have become comparatively less frequent in more recent reports, coinciding with the increasing use of anti-TNF agents. Thalidomide emerged as a pivotal rescue therapy in the early 2000s for corticosteroid-dependent or refractory GI disease. The first pediatric case of complete remission with thalidomide after failure of enteral nutrition, sulfasalazine, azathioprine, and methylprednisolone was reported by Terrin et al. [67]. This approach was subsequently confirmed in two Japanese patients, both achieving sustained remission with steroid discontinuation and growth recovery [68]. Anti-TNF agents (first infliximab, then adalimumab) progressively replaced all these agents as the preferred drug in refractory cases from the mid-2000s onwards. A pediatric case of complete remission induced by infliximab after failure of multiple conventional agents was reported by Ugras et al. [71]. A similar outcome was described by Iwama et al. in a 15-year-old girl with terminal ileum BD who had failed mesalazine, colchicine, corticosteroids, azathioprine, and cyclosporine [72]. The successful use of adalimumab for GI-PedBD, achieving full mucosal healing, was reported by Kaji et al. and Gallizzi et al. [75,76]. In general, GI involvement, along with younger disease onset and elevated inflammatory parameters, may suggest a poorer response to conventional therapies, highlighting the clinical severity of GI-PedBD and supporting the rationale for early biological use. Anti-TNF agents, particularly adalimumab, appear to be the most promising therapeutic option for (refractory) GI-PedBD, as well as (in general) for PedBD patients responding poorly to conventional therapies [78,79,80]. This general and initial evidence in GI-PedBD seems to parallel the broader experience with biologic drugs (with specific reference to anti-TNF agents) across pediatric rheumatology, where anti-TNF agents have substantially transformed the management of other pediatric rheumatic conditions and IBDs in the early phase of disease and/or when refractory to conventional disease-modifying therapy [81,82,83,84].

This systematic review has several limitations that must be acknowledged. The most critical is the paucity of high-quality prospective studies with systematic GI assessment: the vast majority of included studies were retrospective, were not focused on investigating GI involvement in PedBD (indeed, GI endoscopy findings are available for a minority of the selected studies), and relied on heterogeneous GI case definitions (in addition to the heterogeneity of BD diagnostic criteria in general across studies). Furthermore, since this review specifically focused on the pediatric BD population, a systematic comparison with the adult GI-BD literature was beyond its scope. Finally, the lack of an absolute and constant “operational definition of GI involvement” should be also considered in interpreting our findings.

Despite these limitations, this review has important clinical and research implications. Clinically, it raises and/or reinforces the importance of actively screening for GI symptoms in all PedBD patients, since GI involvement could be more frequent than currently thought. Since the manifestations of GI involvement could be quite nonspecific and/or relatively mild, gastroenterological consultation (and, if appropriate) endoscopic evaluation should be considered in PedBD patients. From a research perspective, there is an urgent need for standardized, prospective, multicenter cohort studies that adopt uniform GI assessment protocols, as well as validated and uniform diagnostic criteria in the pediatric population. The development of clear and consolidated diagnostic criteria to define GI-PedBD as well as a disease-specific outcome measure, analogous to those used in pediatric IBDs, would also be a valuable step toward more meaningful and comparable data collection.

## 5. Conclusions

GI involvement in PedBD patients is not negligible. Its frequency across published pediatric cohorts ranges widely, with a median estimate of approximately 15% in unselected populations, and it may be higher in specific geographic or clinical settings.

GI-PedBD is clinically heterogeneous but can potentially cause severe manifestations in children and adolescents. Abdominal pain and diarrhea are the most commonly reported symptoms. The pathological findings are mainly represented by mucosal inflammation of the colon, esophagus, and stomach, where ulcers and erosions are frequently described in GI-PedBD. Currently, anti-TNF agents (especially adalimumab) appear an effective approach for refractory GI-PedBD, even though the evidence is still limited. Prospective, multicenter studies with standardized GI assessment protocols are clearly needed to better characterize this specific subgroup of PedBD patients and optimize their therapeutic management.

## Figures and Tables

**Figure A1:**
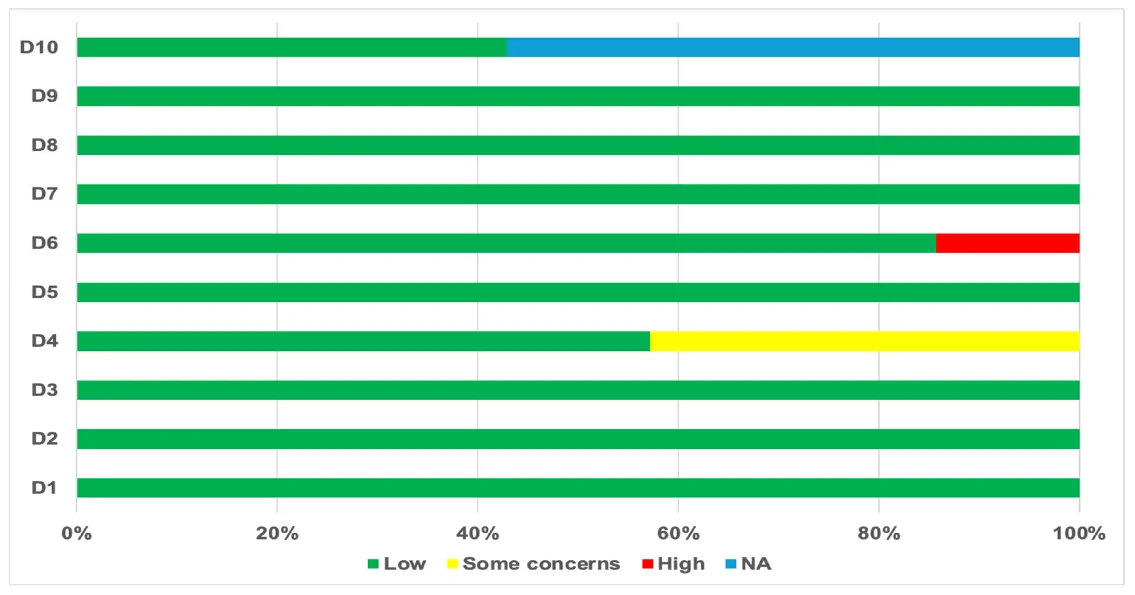
Risk of bias summary for case series.

**Figure A2:**
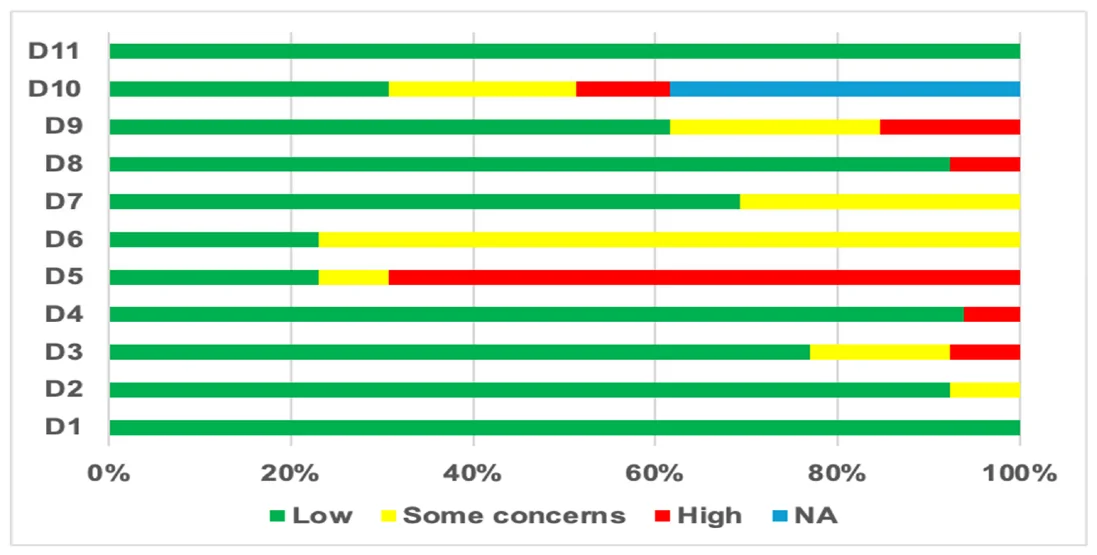
Risk of bias summary for cohort studies.

**Figure A3:**
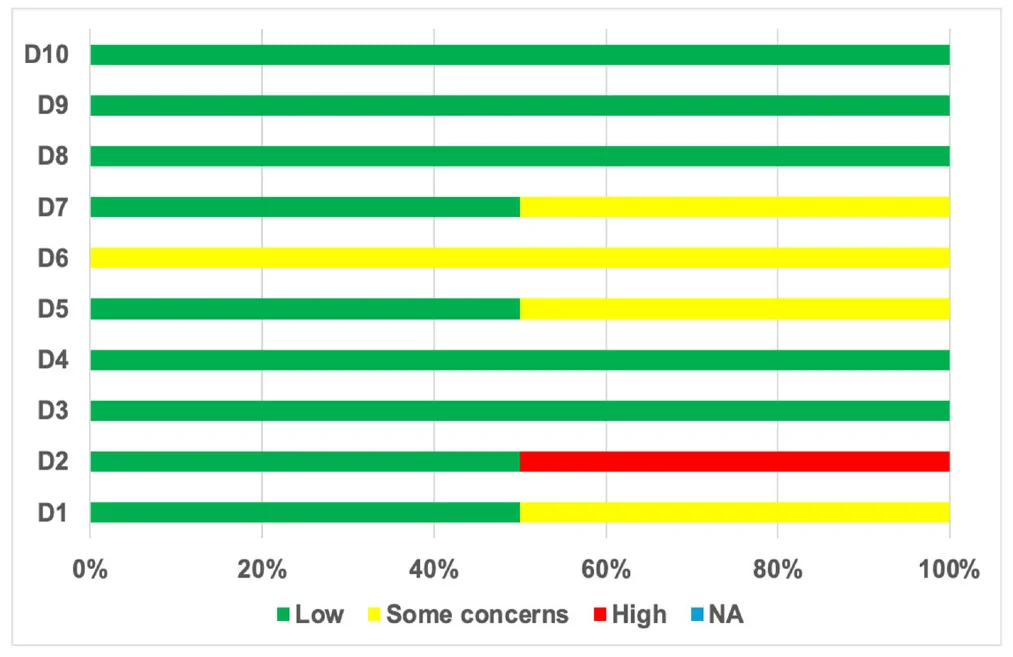
Risk of bias summary for case–control studies.

**Figure A4:**
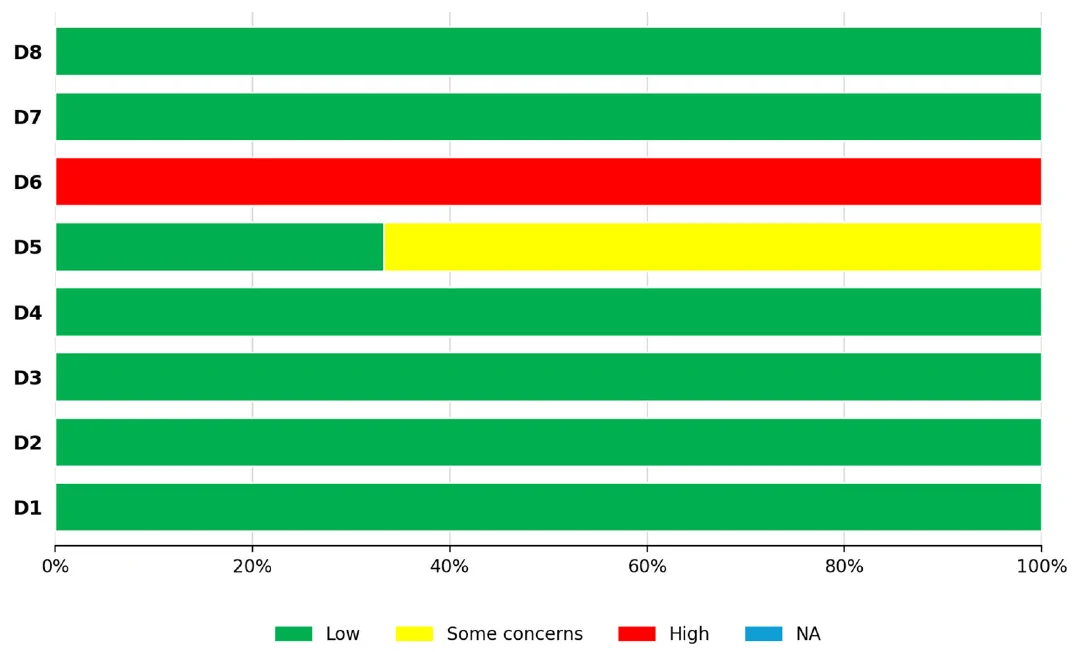
Risk of bias summary for cross-sectional studies.

**Figure A5:**
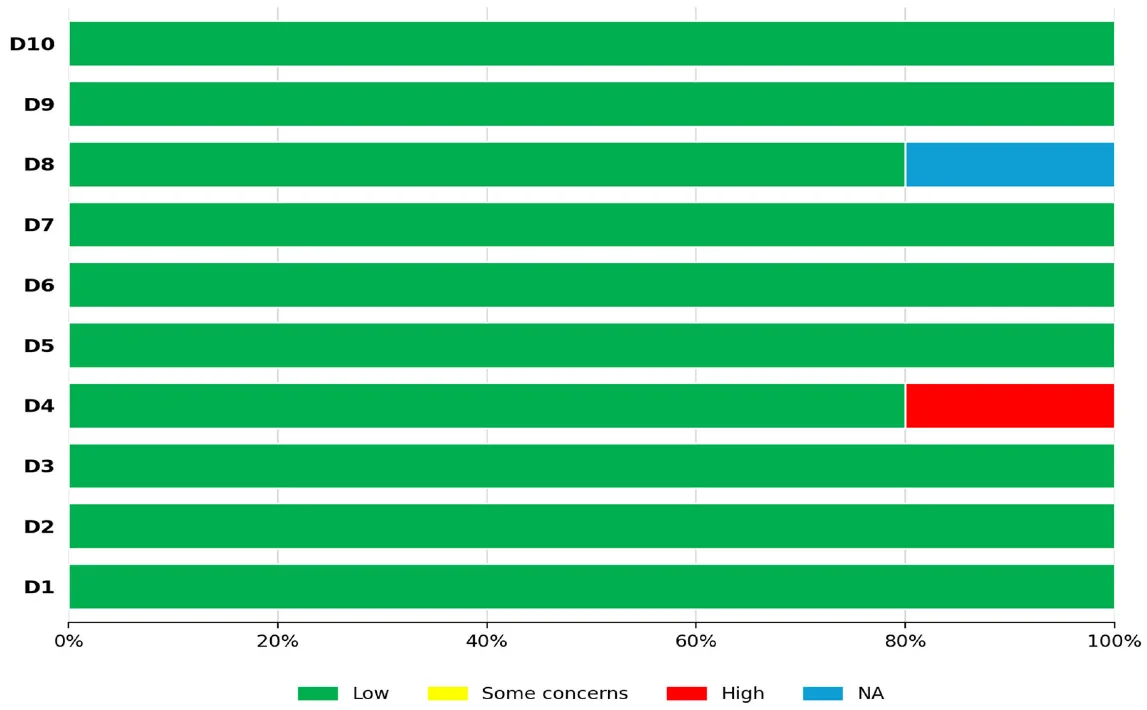
Risk of bias summary for descriptive studies.

## Data Availability

No new data were created or analyzed in this study. Data sharing is not applicable to this article.

## Supplementary Materials

The following supporting information can be downloaded at: https://www.mdpi.com/article/10.3390/diagnostics16193184/s1, Supplementary Table S1: Individual patients with gastrointestinal Behçet’s disease reported in case reports.

## Abbreviations

The following abbreviations are used in this manuscript:

5-ASA	5-aminosalicylic acid (mesalazine/mesalamine)
ADA	adalimumab
AZA	azathioprine
BD	Behçet’s disease
CENTRAL	Cochrane Central Register of Controlled Trials
CINAHL	Cumulative Index to Nursing and Allied Health Literature
COL	colchicine
CS	corticosteroids
CSA	cyclosporine A
CYC	cyclophosphamide
EN	enteral nutrition
ETN	etanercept
GI	gastrointestinal
GI-PedBD	gastrointestinal involvement in pediatric Behçet’s disease
IBD	inflammatory bowel disease
ICBD	International Criteria for Behçet’s Disease
IFX	infliximab
ISG	International Study Group (criteria)
JBI	Joanna Briggs Institute
MMF	mycophenolate mofetil
MPDN	methylprednisolone
MTX	methotrexate
PDN	prednisolone/prednisone
PedBD	pediatric Behçet’s disease
PEDBD	Pediatric Behçet’s Disease (classification criteria)
PRISMA	Preferred Reporting Items for Systematic Reviews and Meta-Analyses
SSZ	sulfasalazine/salazosulfapyridine
TAC	tacrolimus
THAL	thalidomide
TNF	tumor necrosis factor
TPN	total parenteral nutrition

## Appendix A

This Appendix reports, in full, the search strategy applied to each electronic database, as summarized in Section 2.3 (Search strategy).

**Table A1:** Literature search strategies.

Search ID	Search Terms	Output
**MEDLINE (Ovid)** *Ovid MEDLINE^®^ ALL <1946 to 16 December 2024>*		
1	Behcet Syndrome/	(10,451)
2	HLA-B51 Antigen/	(396)
3	(adamantiades behcet disease OR adamantiades-behcet disease OR behcet disease OR behcet’s disease OR behcet syndrome OR behcet’s syndrome OR HLA B51 OR HLA-B51 OR malignant aphthosis OR Silk Road disease OR Silk Route disease OR behcet triple symptom complex OR triple symptom complex OR Behcet ulcer OR Behcet’s ulcer*).mp.	(13,622)
4	or/1–3	(13,622)
5	Infant, Newborn/	(693,889)
6	Infant/	(895,912)
7	Child, Preschool/	(1,021,428)
8	Child/	(1,998,355)
9	Adolescent/	(2,294,772)
10	(pediatric OR paediatric OR neonate* OR newborn* OR infan* OR preschool* OR child OR children OR preadolesc* OR prepubescent OR pubescent OR adolesc* OR juvenile* OR preteen* OR teen* OR young* OR youth* OR pediatric onset OR paediatric onset OR juvenile onset).mp.	(5,875,997)
11	or/5–10	(5,875,997)
12	4 and 11	(3345)
**EMBASE (Ovid)** *Embase Classic + Embase <1947 to 16 December 2024>*		
1	Behcet Disease/	(19,835)
2	HLA B51 Antigen/	(1204)
3	(adamantiades behcet disease OR adamantiades-behcet disease OR behcet disease OR behcet’s disease OR behcet syndrome OR behcet’s syndrome OR HLA B51 OR HLA-B51 OR malignant aphthosis OR Silk Road disease OR Silk Route disease OR behcet triple symptom complex OR triple symptom complex OR Behcet ulcer*).mp.	(23,547)
4	or/1–3	(23,547)
5	Newborn/	(726,853)
6	Infant/	(853,625)
7	Preschool Child/	(734,067)
8	Child/	(2,534,353)
9	Adolescent/	(2,042,853)
10	(pediatric OR paediatric OR neonate* OR newborn* OR infan* OR preschool* OR child OR children OR preadolesc* OR pubescent OR adolesc* OR juvenile* OR preteen* OR teen* OR young* OR youth* OR pediatric onset OR paediatric onset OR juvenile onset).mp.	(6,748,889)
11	or/5–10	(6,748,889)
12	4 and 11	(4872)
13	limit 12 to (conference abstracts or embase or preprints)	(4303)
**CENTRAL (Cochrane)** *EBM Reviews—Cochrane Central Register of Controlled Trials <November 2024>*		
1	Behcet Syndrome/	(171)
2	(adamantiades behcet disease OR adamantiades-behcet disease OR behcet disease OR behcet’s disease OR behcet syndrome OR behcet’s syndrome OR HLA B51 OR HLA-B51 OR malignant aphthosis OR Silk Road disease OR Silk Route disease OR behcet triple symptom complex OR Behcet ulcer*).mp.	(388)
3	or/1–2	(388)
4	Infant, Newborn/	(23,536)
5	Infant/	(31,040)
6	Child, Preschool/	(39,947)
7	Child/	(72,544)
8	Adolescent/	(138,630)
9	(pediatric OR paediatric OR neonate* OR newborn* OR infan* OR preschool* OR child OR children OR preadolesc* OR pubescent OR adolesc* OR juvenile* OR preteen* OR teen* OR young* OR youth* OR pediatric onset OR juvenile onset).mp.	(458,916)
10	or/4–9	(458,916)
11	3 and 10	(78)
**CINAHL Complete (EBSCOhost)** *CINAHL Complete—Advanced Search; Expanders: Apply equivalent subjects; Search modes: Proximity*		
S1	(MH “Behcet’s Syndrome”)	1684
S2	“HLA-B51 Antigen”	2
S3	TX (adamantiades behcet disease OR behcet disease OR behcet’s disease OR behcet syndrome OR behcet’s syndrome OR HLA B51 OR HLA-B51 OR malignant aphthosis OR Silk Road disease OR Silk Route disease OR behcet triple symptom complex OR Behcet ulcer*)	3009
S4	S1 or S2 or S3	3009
S5	(MH “Infant, Newborn”)	149,376
S6	(MH “Infant”)	194,421
S7	(MH “Child, Preschool”)	234,308
S8	(MH “Child”)	540,579
S9	(MH “Adolescence”)	625,255
S10	TX (pediatric OR paediatric OR neonate* OR newborn* OR infan* OR preschool* OR child OR children OR preadolesc* OR pubescent OR adolesc* OR juvenile* OR preteen* OR teen* OR young* OR youth* OR pediatric onset OR juvenile onset)	2,484,228
S11	S5 or S6 or S7 or S8 or S9 or S10	2,484,228
S12	S4 and S11	1166

Databases searched: MEDLINE (Ovid), EMBASE (Ovid), CENTRAL (Cochrane), and CINAHL Complete (EBSCOhost). Search date: 16 December 2024. Truncation symbol (*) was used to retrieve all word endings. mp. = multi-purpose field search (title, abstract, heading word, drug trade name, manufacturer, device trade name, original title, device manufacturer, device trade name, keyword heading, environmental term). MH = subject heading (CINAHL); TX = all text (CINAHL).

## Appendix B

This Appendix reports the full results of the risk-of-bias assessment performed with the Joanna Briggs Institute (JBI) critical appraisal tools, as summarized in Section 2.4 (Quality assessment), for each study design represented among the included studies (case series, cohort studies, case-control studies, cross-sectional studies, and descriptive studies). For each study design, a domain-by-domain assessment table is followed by a graphical summary of the distribution of judgements (Low risk/Some concerns/High risk/not applicable, NA) across the corresponding ROB domains.

**Table A2:** Risk of bias assessment for case series.

	D1	D2	D3	D4	D5	D6	D7	D8	D9	D10	OVERALL
Borlu [18]										-	
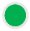
Ekici Tekin [30]											
Krause [15]											
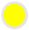
Lv [43]											
Metreau-Vastel [42]										-	
Vignola [17]										-	
Wang X [41]										-	
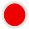

Evaluation: , Low; , Some concerns; , High.

ROB tool domains

D1. Were there clear criteria for inclusion in the case series?
D2. Was the condition measured in a standard, reliable way for all participants included in the case series?
D3. Were valid methods used for the identification of the condition for all participants included in the case series?
D4. Did the case series have consecutive inclusion of participants?
D5. Did the case series have complete inclusion of participants?
D6. Was there clear reporting of the demographics of the participants in the study?
D7. Was there clear reporting of clinical information of the participants?
D8. Were the outcomes or follow-up results of cases clearly reported?
D9. Was there clear reporting of the presenting site(s)/clinic(s) demographic information?
D10. Was the statistical analysis appropriate?

**Table A3:** Risk of bias assessment for cohort studies.

	D1	D2	D3	D4	D5	D6	D7	D8	D9	D10	D11	OVERALL
Boluk [36]										-		
Bozkurt [37]												
Demir [31]										-		
Gaggiano [32]												
Gezgin Yildirim [27]										-		
Kone-Paut [20]												
Kone-Paut [21]												
Maccora [39]												
Mastrolia [34]										-		
Nanthapisal [22]												
Ostrovsky [35]										-		
Pain [28]										-		
Shahram [24]										-		

Evaluation: , Low; , Some concerns; , High.

ROB tool domains

D1. Were the two groups similar and recruited from the same population?
D2. Were the exposures measured similarly to assign people to both exposed and unexposed groups?
D3. Was the exposure measured in a valid and reliable way?
D4. Were confounding factors identified?
D5. Were strategies to deal with confounding factors stated?
D6. Were the groups/participants free of the outcome at the start of the study (or at the moment of exposure)?
D7. Were the outcomes measured in a valid and reliable way?
D8. Was the follow up time reported and sufficient to be long enough for outcomes to occur?
D9. Was follow up complete, and, if not, were the reasons to loss to follow up described and explored?
D10. Were strategies to address incomplete follow up utilized?
D11. Was appropriate statistical analysis used?

**Table A4:** Risk of bias assessment for case–control studies.

	D1	D2	D3	D4	D5	D6	D7	D8	D9	D10	OVERALL
Gungorer [33]											
Mastrolia [40]											

Evaluation: , Low; , Some concerns; , High.

ROB tool domains

D1. Were the groups comparable other than the presence of disease in cases or the absence of disease in controls?
D2. Were cases and controls matched appropriately?
D3. Were the same criteria used for identification of cases and controls?
D4. Was exposure measured in a standard, valid and reliable way?
D5. Was exposure measured in the same way for cases and controls?
D6. Were confounding factors identified?
D7. Were strategies to deal with confounding factors stated?
D8. Were outcomes assessed in a standard, valid and reliable way for cases and controls?
D9. Was the exposure period of interest long enough to be meaningful?
D10. Was statistical analysis appropriate?

**Table A5:** Risk of bias assessment for cross-sectional studies.

	D1	D2	D3	D4	D5	D6	D7	D8	OVERALL
Butbul-Aviel [26]									
Zou [29]									
Galizzi [23]									

Evaluation: , Low; , Some concerns; , High.

ROB tool domains

D1. Were the criteria for inclusion in the sample clearly defined?
D2. Were the study subjects and the setting described in detail?
D3. Was the exposure measured in a valid and reliable way?
D4. Were objective, standard criteria used for measurement of the condition?
D5. Were confounding factors identified?
D6. Were strategies to deal with confounding factors stated?
D7. Were outcomes assessed in a standard, valid and reliable way for cases and controls?
D8. Was appropriate statistical analysis used?

**Table A6:** Risk of bias assessment for descriptive studies.

	D1	D2	D3	D4	D5	D6	D7	D8	D9	D10	OVERALL
Hu [25]											
Hu [38]											
Karincaoglu [19]											
Vaiopoulos [16]											
Kone-Paut [14]											

Evaluation: , Low; , Some concerns; , High.

ROB tool domains

D1. Were there clear criteria for inclusion in the study?
D2. Was the condition measured in a standard, reliable way for all participants included?
D3. Were valid methods used for identification of the condition for all participants included?
D4. Did the study have consecutive inclusion of participants?
D5. Did the study have complete inclusion of participants?
D6. Was there clear reporting of the demographics of the participants in the study?
D7. Was there clear reporting of clinical information of the participants?
D8. Were the outcomes or follow-up results clearly reported?
D9. Was there clear reporting of the presenting site(s)/clinic(s) demographic information?
D10. Was statistical analysis appropriate?
