# Supplementary material for: Gastrointestinal Involvement in Pediatric Behçet’s Disease: A Focused Analysis from a Comprehensive Systematic Review
Source: Diagnostics (Basel). 2026 Sep 30;16(19):3184. doi: 10.3390/diagnostics16193184 (PMC13664234; doi:10.3390/diagnostics16193184)
Supplement: Supplementary file 1 [file diagnostics-16-03184-s001.zip › diagnostics-4528157-supplementary.pdf]

SUPPLEMENTARY MATERIAL

Supplementary Table S1. Individual patients with gastrointestinal Behçet's disease reported in case reports.

First author	Year	Country	Age (GI onset) Gender	BD Diagnostic criteria	GI symptoms	GI lesions	GI disease location	Compli-cations (if any)	Drugs (dose)	Surgery (if any)	Follow-up	GI outcome	Notes
Stringer [62]	1986	Canada	10 y M	n/a	Abdominal pain	Mucosal inflammation; pseudo-polyps; linear ulcers	Ileocecal region	Multiple bowel fistulae	CS (n/a); SSZ (n/a); COL (n/a)	Right ileo-colec-tomy	2 y	Persistent disease after surgery	*- Non-GI: nasal septum perforation - Disease persisted post-surgery*
Stringer [62]	1986	Canada	11 y F	n/a	Abdominal pain	Mucosal inflammation nodularity (lymphoid hyperplasia)	Terminal ileum	None	n/a	None	n/a	Improved	*- Non-GI: oral and genital mucosal lesions; arthritis; skin le-sions*
Stringer [62]	1986	Canada	4 y F	n/a	Abdominal pain	Mucosal ulcerations	Rectum; colon; terminal ileum	None	n/a	None	1 y	Progres-sive disease	*- Non-GI: oral and genital aph-thae; arthritis; skin lesions. - Progressive ter-minal ileal in-volvement at the follow-up*
Stringer [62]	1986	Canada	6 mo M	n/a	Diarrhea; perianal ulcerations	Mucosal ulcerations; fo-cal nodularity (lymphoid hyperplasia)	Rectum; terminal ileum	None	n/a	None	18 mo	Stable	*Non-GI: recur-rent conjunctivi-tis*

Chong [63]	1988	United Kingdom	2 mo M	Japanese BD Research Committee	Diarrhea (bloody); abdominal distension; perianal ulcerations	Mucosal ulcerations	Colon	Perianal abscess	SSZ (n/a); PDN (n/a); AZA (n/a); TPN	Subtotal colectomy + ileostomy; ileorectal anastomosis; colostomy	Until 6 y of age	persistent perianal disease; growth retardation	*- Non-GI: oral aphthous ulcerations<br>- Multiple surgical interventions needed*
Chong [63]	1988	UK	5 wk F	Japanese BD Research Committee	Diarrhea (bloody); perianal ulcerations	Mucosal ulcerations	Colon	None	SSZ (n/a); CS (n/a); TPN	Subtotal colectomy + ileostomy	Until 3.5 y of age	Persistent diarrhea	*- Sister of previous case<br>- Non-GI: oral aphthous ulcerations. Persistent growth failure post-colectomy*
Tolia [64]	1989	USA	15 y M	n/a	Abdominal pain; diarrhea; weight loss	Linear ulcers; pseudo-polyps; mucosal granulomas	Colon; cecum; terminal ileum	None	PDN (n/a); SSZ (n/a)	None	Several months	Relapsed disease	*- Non-GI: anterior and posterior uveitis; erythema nodosum; arthralgia; oral, penile and perianal ulcers<br>- Disease relapsed on steroid tapering*
Tabata [65]	1999	Japan	5 y M	n/a	Abdominal pain	Mucosal ulcerations	Cecum; terminal ileum	Perforation of terminal ileum	Elemental diet; TPN; CS (n/a); CSA (n/a)	Subtotal colectomy + ileostomy (age 6); surgery for	Until death	Fatal	*- Death at 12 yrs of age (viral pneumonia and multiple organ failure)<br>- Non-GI: genital ulcer and erythema nodosum;*

										terminal ileum perforation (age 9)			*severe neurological picture (dysarthria, tremor, gait disturbance), superior vena cava syndrome*
Yamazaki [66]	1999	Japan	6 y F	Japanese BD Research Committee	Lower abdominal pain; positive fecal occult blood	Colitis	Descending colon	None	CS (n/a)	None	2 y	Recurring perianal ulcers	*- Non-GI: recurrent oral aphthae; genital and perianal ulcers; folliculitis-like erythema*<br>*- GI symptoms were self-limited; no endoscopy performed*
Terrin [67]	2002	Italy	13 y F	ISG	Abdominal pain; diarrhea with mucus; hematochezia	Mucosal ulcers and inflammation	Ileum; colon	None	THAL (100-200 mg/d); EN; SSZ (n/a); AZA (n/a); MPDN (n/a)	None	2 y	Complete remission	*- Non-GI: sacroiliitis episcleritis, keratoconjunctivitis*<br>*- Complete remission with THAL after failure of conventional therapy*
Yasui [68]	2003	Japan	9 y M	Japanese BD Research Committee	Abdominal pain; perianal ulcerations	Mucosal ulcerations	Ileocecum	Intestinal perforation with peritonitis (age: 14 yrs)	PDN (10-20 mg/d); THAL (2.5 mg/kg/d, then reduced)	Surgical resection (perforated peritonitis)	>1 y (post-THAL)	Sustained remission;	*- Non-GI: oral/genital/perianal ulcers; erythematous and vesicular skin lesions*<br>*- Steroid-induced osteoporosis with vertebral fracture.*<br>*- Sustained*

													remission on THAL with growth recovery
Yasui [68]	2003	Japan	12 y F	Japanese BD Research Committee	Abdominal pain	Mucosal inflammation	Ileocecum	None	SSZ (2 g/d); PDN (0.5-2mg/kg/d) CSA (n/a); COL (n/a); THAL (2.5 mg/kg/d, then reduced)	None	>4 mo (post-THAL)	Sustained remission	*- Non-GI: oral and genital ulcers; erythema nodosum;* *- Steroid-induced diabetes mellitus resolved after steroid with-drawal following THAL initiation*
Wu [69]	2005	Taiwan	2 wk M	Japanese BD Research Committee	Chronic diarrhea	Mucosal ulcerations	Colon	None	PDN (15 mg/d, ta-pered over 4 months)	None	3 y	Recurrent disease	*- Non-GI: oral ulcers; perianal pustulosis; macu-lopapular skin rash; erythematous nodules; growth retardation; anemia Recurrent epi-sodes responding to short-term PDN*
Nakamura [70]	2006	Japan	16 y F	Japanese BD Research Committee	Abdominal pain; melena	Mucosal inflammation and ulcers	Colon; ileum	Perito-neal irritation	PDN (20 mg/d)	Total co-lectomy + partial ileectom y + ileo-rectal anasto-mosis	Post-opera-tive	n/a	*- Non-GI: oral and genital aphthae; pyo-derma gangreno-sum on the left lower leg (re-solved after colec-tomy)*

Ugras [71]	2008	Turkey	5 y F	ISG	Recurrent diarrhea (mucous); abdominal pain	Mucosal inflammation Mucosal ulceration	Rectum; colon; ileocecal valve; terminal ileum	None	5-ASA (40 mg/kg/d); COL (0.02 mg/kg/d); PDN (0.2 mg/kg/d); AZA (2.3 mg/kg/d); IFX (5 mg/kg at wk 0, 2, 6)	None	7 mo post-IFX	Complete remission	*- Non-GI: oral ulcers; genital ulcers; arthralgia; isolated seizures - Remission after 3rd IFX dose with ulcers healed and no diarrhea; weight gain*
Iwama [72]	2011	Japan	15 y F	ISG	Right lower quadrant abdominal pain	Mucosal ulcerations	Terminal ileum; ascending colon	None	5-ASA (500 mg TID); COL (0.5 mg BID); PDN (15 mg TID); AZA (50 mg/d); CSA (2 mg/kg BID); IFX (wk1:4 mg/kg; W2&wk7: 6 mg/kg)	None	3 mo post-IFX	Complete remission	*- Non-GI: oral ulcer; erythema nodosum, vulvar ulceration. - PDN and AZA discontinued after 2nd infusion: no benefit. Remission after IFX*
Zhang [73]	2013	China	8 y F	ISG	Abdominal pain; nausea; vomiting	Mucosal ulcerations; macroscopic perforations	Esophagus; stomach; ileum; colon (especially ileocecal area)	Intestinal perforations (2 episodes)	PDN (20 mg/d); MTX (5 mg/wk, oral)	Simple perforation repair (1st episode); ileo-colectomy (2nd episode)	Post-operative	Good recovery	*Non-GI: recurrent oral ulcers, genital ulcers, anterior uveitis; - Ileo-colectomy was considered definitive treatment; no recurrence reported*

Watanabe [74]	2013	Japan	5 y F	ISG	Abdominal pain	Aphthous lesions	Transverse colon	None	PDN (15–20 mg/d); COL (0.5 mg BID); IFX (n/a); TAC (1.5 mg/d); ETN (n/a)	None	n/a	Remission	*- Non-GI: recurrent fever; ulcerative gingivostomatitis ; erythema nodosum - Remission with ETN*
Kaji [75]	2015	Japan	13 y F	ISG	Abdominal pain; bloody diarrhea weight loss	Mucosal ulcerations; mucosal inflammation	Terminal ileum	None	TPN; MPDN pulse (1g/d x 3 days); ADA (wk1:160 mg; wk2: 80 mg; 40 mg q2wk)	None	8 mo	Complete remission	*- Non-GI: recurrent oral aphthosis recurrent vulvar aphthosis; arthralgias; erythema nodosum-like eruptions (on the lower extremities); neutrophilic dermatitis - Complete remission under ADA*
Gallizzi [76]	2017	Italy	12 y F	ICBD and PedBD	Abdominal pain; rectal bleeding; diarrhea; nausea	Mucosal aphthae and ulcerations; mucosal inflammation; pseudo-polyps	Stomach; terminal ileum	None	MPDN IV (n/a; 3 days); PDN (n/a); CYC (n/a)-MMF (n/a); ADA (n/a)	elective terminal ileum resection	18 mo (until planned surgery)	Recurrent GI symptoms	*- Non-GI: neuro-BD (pseudotumor cerebri; papilledema; retinal vasculitis); visual loss; recurrent oral aphthosis - Relapsing GI symptoms after ADA, then elective surgery*
Zhang [77]	2021	China	9 y M	ICBD and PedBD	Abdominal pain;	Mucosal inflammation	Terminal ileum;	Perianal abscess	5-ASA (n/a); IFX (n/a);	Appendectomy	2 yrs	GI	*- Non-GI: cerebral venous sinus*

			anal fistula	and ulceration; pseudo-polyps	ileocecal valve	anal fis-tula	THAL (n/a); PDN (low dose); CYC (n/a)	(pre-BD diagno-sis); peri-anal ab-scess drainage		improve-ment	*thrombosis (neuro-BD); uve-itis in the right eye*

Abbreviations: 5-ASA = 5-aminosalicylic acid (mesalazine/mesalamine); ADA = adalimumab; AZA = azathioprine; BID = twice daily; COL = colchicine; CS = corticosteroids (dose not specified); CSA = cyclosporine A; CYC = cyclophosphamide; EN = enteral nutrition; ETN = etanercept; IFX = infliximab; MMF = mycophenolate mofetil; MPDN = methylprednisolone; MTX = methotrexate; n/a = not specified; PDN = prednisolone/prednisone; SSZ = sul-fasalazine/salazosulfapyridine; TAC = tacrolimus; THAL = thalidomide; TPN = total parenteral nutrition; wk(s) = week(s).
